# Enhanced Adsorption of Selenium Ions from Aqueous Solution Using Iron Oxide Impregnated Carbon Nanotubes

**DOI:** 10.1155/2017/4323619

**Published:** 2017-05-07

**Authors:** Omer Y. Bakather, Ahmad Kayvani Fard, Ihsanullah, Majeda Khraisheh, Mustafa S. Nasser, Muataz Ali Atieh

**Affiliations:** ^1^Department of Chemical Engineering, Jazan University, P.O. Box 706, Jazan 45142, Saudi Arabia; ^2^Department of Chemical Engineering, Hadhramout University, Hadhramout, Yemen; ^3^Qatar Environment and Energy Research Institute, Hamad Bin Khalifa University, Qatar Foundation, P.O. Box 5825, Doha, Qatar; ^4^College of Science and Engineering, Hamad Bin Khalifa University, Qatar Foundation, P.O. Box 5825, Doha, Qatar; ^5^Department of Chemical Engineering, King Fahd University of Petroleum & Minerals, Dhahran 31261, Saudi Arabia; ^6^Chemical Engineering Department, Qatar University, P.O. Box 2713, Doha, Qatar; ^7^Gas Processing Centre, College of Engineering, Qatar University, P.O. Box 2713, Doha, Qatar

## Abstract

The aim of this research was to investigate the potential of raw and iron oxide impregnated carbon nanotubes (CNTs) as adsorbents for the removal of selenium (Se) ions from wastewater. The original and modified CNTs with different loadings of Fe_2_O_3_ nanoparticles were characterized using high resolution transmission electron microscopy (HRTEM), scanning electron microscopy (SEM), X-ray diffractometer (XRD), Brunauer, Emmett, and Teller (BET) surface area analyzer, thermogravimetric analysis (TGA), zeta potential, and energy dispersive X-ray spectroscopy (EDS). The adsorption parameters of the selenium ions from water using raw CNTs and iron oxide impregnated carbon nanotubes (CNT-Fe_2_O_3_) were optimized. Total removal of 1 ppm Se ions from water was achieved when 25 mg of CNTs impregnated with 20 wt.% of iron oxide nanoparticles is used. Freundlich and Langmuir isotherm models were used to study the nature of the adsorption process. Pseudo-first and pseudo-second-order models were employed to study the kinetics of selenium ions adsorption onto the surface of iron oxide impregnated CNTs. Maximum adsorption capacity of the Fe_2_O_3_ impregnated CNTs, predicted by Langmuir isotherm model, was found to be 111 mg/g. This new finding might revolutionize the adsorption treatment process and application by introducing a new type of nanoadsorbent that has super adsorption capacity towards Se ions.

## 1. Introduction 

 Selenium (Se) is unique nonmetal chemical element with five known oxidative states under the form of elemental selenium Se^0^, namely, 0, ^−^1, ^−^2, ^+^4, and ^+^6 [1]. Although it is an important trace element for many organisms, exceeding homeostatic levels is considered toxic. Se is typically found on the earth's crust, rocks, and sedimentary soils [2]. Recent reports state that a significant amount of Se emissions into the atmosphere and aquatic environment is related to industrial and mining activities in addition to agricultural drainage run-off [3–5].

Tan et al. [6], in a recent extensive review, reported the different types of organic and inorganic Se species available. The inorganic form of Se is typically found in surface and ground water in a number of reported forms such as Se^−2^ (selinide), SeO_3_^−2^ (selenite), SeO_4_^−2^ (selenate), and Se^0^, which is the species nonsoluble form. If found in high concentration levels in waste or surface water, Se can cause serious environmental problems [7, 8]. The maximum level for Se in surface water was set at 5.0 *μ*g/L by the United States Environmental Protection Agency (USEPA) in 1999. Therefore, due to the increased interest in Se treatment and the more stringent environmental consent levels for its concentration in surface water, the value set in 1999 is being updated by USEPA in 2014 [1, 5, 9]. Furthermore, there is variation in the maximum allowable levels of Se in drinking water worldwide. According to the World Health Organization [10] the value is set at 40 *μ*g/L, while the European Union (EU) sets the Se levels at a much lower value of 10 *μ*g/L [11]. In order to meet the drinking water standards for Se in addition to treating industrial, mining wastewaters and agricultural run-offs, a varied number of treatment processes are reported in the open literature with various degrees of complexity and advancement. Comprehensive reviews conducted by Robberecht and Van Grieken [4] and Tan et al. [6] offer an excellent overview of all current known treatments used for Se. It can be seen from the two recent reviews that although a great variety of biological [12–14], chemical [15–20], and physical treatments technologies [9, 21, 22] were developed in recent years, no single treatment offers a complete and cost effective scheme for treating Se from waste or drinking water. While biological treatments stand as one of the best options for treating Se, challenges related to long-term stability of the biogenic selenium and the predicting of the fate of the bioreduced Se in the environment are two major concerns. On the other hand, adsorption has been reported as an effective lower cost treatment technology for Se removal [23]. Varied adsorbents have been tested including low cost alternative materials [4], activated carbons, and other metal-oxide-enhanced materials. Of the latter, metal-oxide-enhanced materials with ferrihydrite have been reported as the most promising materials for the treatment of water containing SeO_3_^−2^ ions [16]. Reports showed that the strong affinity of SeO_3_^−2^ to iron hydroxide surfaces resulted in around 99% removal rate for a wide pH range (3–8). In contrast, the process was not effective for SO_4_^−2^ and its presence acted as a competitor for the desorption sites causing suppressed removal rates [20]. The inadequate SO_4_^−2^ removal was also related to interacting between the various Se species and the surface of the adsorbent. While SeO_3_^−2^ was reported to form inner-sphere adsorption on iron hydroxide adsorbents, SO_4_^−2^, on the other hand, was adsorbed in an outer sphere manner resulting in an ineffective removal [16]. Accordingly, the development of a process to remove effectively most of the possible inorganic forms of Se is still required. With this in mind and due to their high surface areas, recent studies reported the use of various nanomaterials for the removal of the Se oxyanions. Example such as hydrophilic magnetic nanoparticle-graphene oxide composite was reported recently by Fu et al. [16]. A two-step reaction was used in the preparation of the adsorbents in addition to deposition of iron oxide nanoparticles at high temperatures. The authors reported a maximum removal of 95% of Se (VI) ions at pH ~ 2. While it is not easy to compare the maximum capacity of an adsorbent for the Se removal given the varied preparation methods, costs, and experimental condition, a recent study using a new iron oxide nanoparticles reported a higher removal rates compared with literature reported values [18]. Having said that, the values are still small and the search for other adsorbents is still required for cost effective Se removal from water.

Accordingly, this study is focused on developing multiwalled carbon nanotubes based novel materials for effective removal of Se from aqueous solutions. In recent years, multiwalled carbon nanotubes (CNTs) have been explored widely due to their unique properties [24–26] and have been extensively used in nanotechnology, optics, electronics, material science, and water treatment [27]. CNTs were also reported for the adsorption of heavy metal ions [28–32] and organic compounds [33–36]. Despite the most recent comprehensive review articles and to the best of our knowledge, no studies were reported on the use of multiwalled carbon nanotubes for Se removal. In this work, iron oxide impregnated CNTs were employed for the adsorption of the selenium ions from water. The effect of iron oxide loading, pH, dosage of CNTs, contact time, and initial concentration were studied on the removal of selenium. The raw CNTs and impregnated CNTs were characterized using field emission scanning electron microscopy (FE-SEM), high transmission electron microscopy (HR-TEM), thermogravimetric analysis (TGA), X-ray diffractometer (XRD), energy dispersive X-ray spectroscopy (EDS), Brunauer, Emmett, and Teller (BET) nitrogen adsorption technique, and zeta potential. The adsorption equilibrium data were correlated by the Langmuir and Freundlich isotherms and the kinetic data were analyzed using two kinetic models.

## 2. Experimental

### 2.1. Materials

All solvents used in this study were of analytical grade and purchased from Sigma-Aldrich Co. Ltd. Ethanol liquid (98%, purity) was used as a solvent and iron (III) nitrate as a precursor of iron nanoparticles and selenium dioxide (SeO_2_) was used as source of selenium ions in the water.

### 2.2. Production of Carbon Nanotubes

Floating catalyst chemical vapor deposition reactor was used for the production of CNTs. The experimental setup used and reaction conditions are reported previously by Fard et al. [26]. Briefly, injected vertical chemical vapor deposition (FC-CVD) with quartz tube 100 mm in diameter and 1200 mm in length with flanges fixed at both ends was used to synthesis CNTs. Xylene was used as source of hydrocarbon and the argon gas was used to flush the air from the system, while the hydrogen gas was used as a carrier and reacting gas. Purity of CNTs produced was >96%.

### 2.3. Impregnation of CNTs

The iron oxide nanoparticles were impregnated on the surface of CNTs by a wet impregnation method. For 5% iron oxide loadings, 361 mg of Iron (III) nitrate nonahydrate and 1 g of CNTs were dissolved separately in ethanol solution and sonicated for 30 minutes to ensure uniform mixing. Upon further mixing of the two solutions and further sonication, the solution was kept in a furnace at 80°C overnight to evaporate the ethanol. Finally, the product was calcined at 350°C for 3 hours in the convection oven to ensure effective attachment of the iron oxide particles onto the surface of CNTs. After cooling, the composite of CNTs with 5% iron oxide NP is synthesized. To produce CNTs with 10% and 20% iron oxide loading, 722 mg and 1.44 g Iron (III) nitrate nonahydrate are mixed with 1 g of CNTs, respectively. Details of the preparation are found elsewhere [37].

### 2.4. Characterization of Carbon Nanotubes

#### 2.4.1. Crystal Structure

Powder X-ray diffraction (XRD) patterns were recorded using a Rigaku MiniFlex-600. The X-ray diffractometer with Cu K*α* radiation *λ* = 1.54 Å at a rate of 0.4% over Bragg angles ranging from 10 to 90° was used for the analysis. The operating voltage and current were maintained at 40 kV and 15 mA, respectively.

#### 2.4.2. Surface Structure

Scanning electron microscopy (SEM) was performed using FEI Quanta 200 Environmental Scanning Electron Microscope (ESEM) with a resolution of 5 nm and magnification 200K to observe the morphology and structure of the material. Also, the morphological and structural analysis of CNTs was conducted using transmission electron microscopy (TEM) (CM12, Philips).

#### 2.4.3. Point of Zero Charge

To measure the surface charge and zeta potential Zetasizer (Nano ZS 90, Malvern Instruments Ltd., Malvern, UK) equipped with a 4.0 mW internal laser was used. The Zetasizer works on the principle of dynamic light scattering (DLS). The measurements were performed at room temperature (25°C) with a scattering angle of 90°.

#### 2.4.4. Other Chemical Properties and Surface Area

To analyze physical and chemical properties of CNTs with respect to temperature, the thermogravimetric analyses were performed using a TGA analyzer (SDT, Q600) at a heating rate of 10°C/min in air. The surface areas of CNTs were measured by N_2_ adsorption at 77 K using BET surface area analyzer (Micromeritics ASAP 2020).

### 2.5. Preparation of the Selenium Stock Solution

Specific amount of SeO_2_ was dissolved in deionized water to prepare the stock solution. SeO_2_ dissolves in water to form selenous acid (SeO_2_^−3^). The pH of the stock solution was adjusted by using 0.1 M NaOH or 0.1 M HNO_3_ and maintained by the addition of buffer solutions.

### 2.6. Se Sorption Experiments

In order to assess the effectiveness of the new adsorbent, batch adsorption experiments were conducted at room temperature in 1 L glass beakers. 50 mL of selenium solution of required concentration was placed in the flasks, covered, and mounted on the mechanical rotary shaker (MPI Lab Shaker) to ensure adequate mixing. Different experimental runs were conducted to study the effect of solution pH, contact time, CNTs dosage, and initial Se (IV) ions concentration on the adsorption of Se ions. Inductively coupled plasma mass spectroscopy (ICP-MS) was employed to analyze the concentrations of the samples. The adsorption capacity (*Q*) and removal efficiency (RE) were calculated as follows [37]:

(1) Q=Ci−Cf×VWgRE%=Ci−CfCi×100,

where *C*_*i*_ (mg/L) is the initial concentration of selenium in the water, *C*_*f*_ (mg/L) is the final concentration of the selenium in the water, *V* (L) is the volume of the water, and *W*_*g*_ is the mass of CNTs.

### 2.7. Adsorption Isotherms Models

The Langmuir and Freundlich isotherms were used to study the adsorption performance and to determine the adsorption capacity for the adsorbent [37]. The Langmuir adsorption isotherm is expressed as follows:

(2) $$\begin{array}{c} Q_{e}=\frac{q_{m}K_{L}C_{e}}{1+K_{L}C_{e}}, \end{array}$$

where *Q*_*e*_ (mg/g) and *q*_*m*_ (mg/g) are the amount adsorbed at equilibrium and the maximum adsorption capacity, respectively, while *C*_*e*_ (mg/L) is the equilibrium adsorbate concentration and *K*_*L*_ is Langmuir constant.

Equation (2) can be linearized as follows:

(3) $$\begin{array}{c} \frac{C_{e}}{Q_{e}}=\frac{C_{e}}{q_{m}}+\frac{1}{K_{L}q_{m}}. \end{array}$$

Freundlich isotherm is expressed as follows:

(4) $$\begin{array}{c} Q_{e}=K_{f}C_{e}^{1/n}. \end{array}$$

Equation (4) can be linearized as follows:

(5) $$\begin{array}{c} \log Q_{e}=\frac{1}{n}\log C_{e}+\log K_{f}, \end{array}$$

where *n* and *K*_*f*_ are the empirical constants.

### 2.8. Adsorption Kinetics

In order to find the maximum selenium removal by CNTs and to model the experimental data, two well-known kinetic models, pseudo-first-order and pseudo-second-order models, were used in this study.

The Lagergren pseudo-first-order model proposes that the rate of sorption is proportional to the number of sites unoccupied by the adsorbate [37]. The linear form of the pseudo-first-order equation can be expressed as follows:

(6) $$\begin{array}{c} \ln\left(\;Q_{e}-Q_{t}\;\right)=\ln Q_{e}-k_{1}t, \end{array}$$

where *Q*_*t*_ is the sorption capacity (mg/g) at any preset time interval (*t*) and *k*_1_ is the pseudo-first-order rate constant (min^−1^). A graph of ln⁡(*Q*_*e*_ − *Q*_*t*_) versus time is plotted and the constant is found. Additionally, the adsorption data were modeled to the pseudo-second-order kinetic model [37] where its linear form is expressed as follows:

(7) $$\begin{array}{c} \frac{t}{Q_{t}}=\frac{1}{k_{2}Q_{e}^{2}}+\frac{t}{Q_{e}}, \end{array}$$

where *k*_2_ is the pseudo-second-order rate constant (g/mg·min). By plotting *t*/*Q*_*t*_ versus time, straight lines were obtained and the constants, *k*_2_ and *Q*_*e*_, were found.

## 3. Results and Discussion

### 3.1. Characterization of Carbon Nanotubes

**Figure 1:**
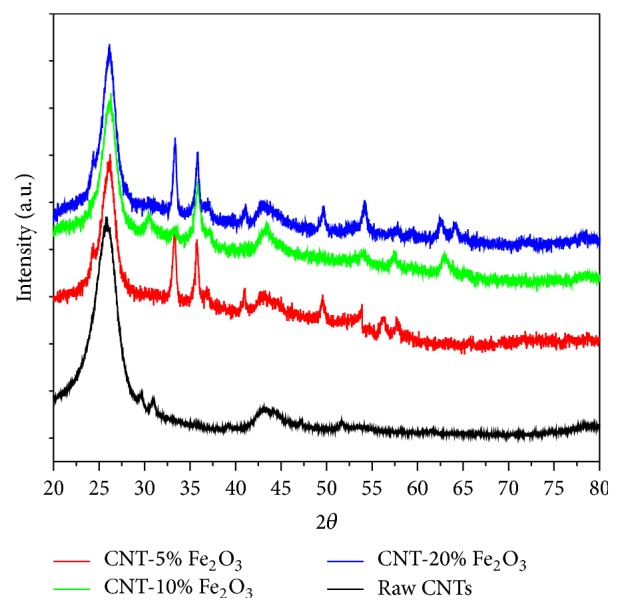
XRD analysis of raw CNTs, and CNT-5% iron oxide, CNT-10% iron oxide, and CNT-20% iron oxide.

Figure 1 shows the X-ray diffraction patterns of CNTs and impregnated CNTs with Fe_2_O_3_ nanoparticles. The XRD diffraction pattern of pure *α*-Fe_2_O_3_ is similar to that of CNTs impregnated Fe_2_O_3_ nanoparticles confirming the presence of *α*-Fe_2_O_3_ crystal nanoparticles on the surfaces of CNTs. The distinct peaks of *α*-Fe_2_O_3_ crystalline structure are found at 2*θ* of 34, 36, 42, 50, 54, 63, 65, 72, and 75. The characteristic peak of CNTs was observed at 2*θ* of 27 which corresponds to C (002) and indicative of proper and undamaged graphite structure of the CNTs. The other characteristic diffraction peaks of graphite are at 2*θ* of 43°, 45°, and 77° and associated with C (100), C (101), and C (110) diffractions of graphite, respectively. Peaks indexed at C (002), C (100), and C (101) are indication of hexagonal structure of CNTs and the presence of C (002) peak in the XRD data confirms the multiwalled nature of the carbon nanotubes [42].

The sharp peak at the 2*θ* of 25.5° of the raw and impregnated CNTs is indicative of undamaged (no impurities in lattice) and developed graphite structure. Defects would have been observed in the CNTs if the 2*θ* peak of 25.5° was broader and a shift of the peak diffraction towards lower angles was detected. Peaks at 31, 44, and 52.5 are indicative of the multiwalled nature of the carbon nanotubes. The results observed by the peaks in Figure 1 are a clear testament to the highly crystalline, uniform, highly ordered, and pure CNTs. The results here are further confirmed by SEM and TEM images.

In addition to XRD, the raw and impregnated CNTs were characterized further using field-scanning electron microscopy (FE-SEM), high resolution transmission electron microscopy (HR-TEM), thermogravimetry (TGA) techniques, XRD, BET surface area, and zeta potential.

**Figure 2:**
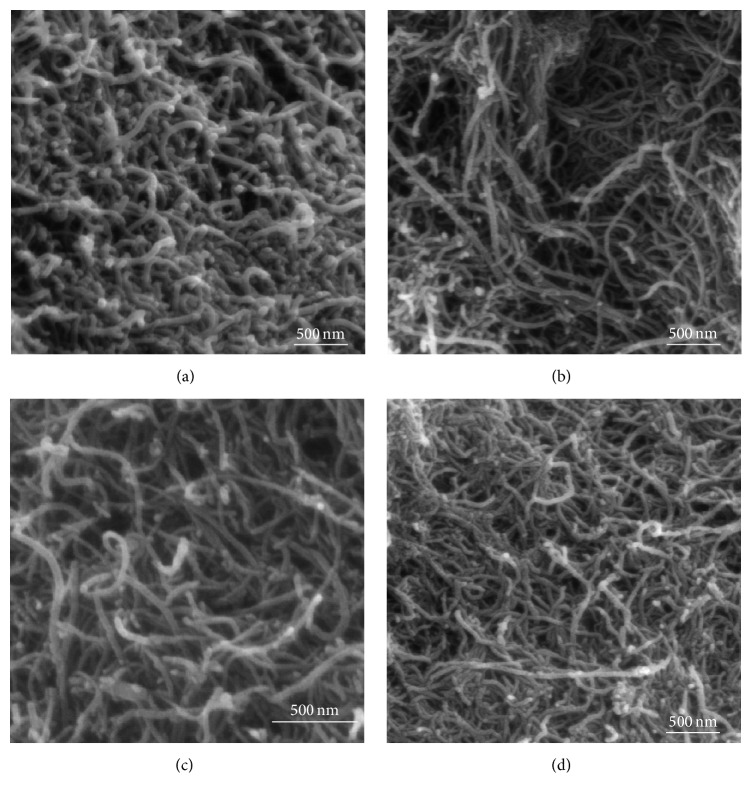
SEM images of (a) raw CNTs, (b) CNT-5% iron oxide, (c) CNT-10% iron oxide, and (d) CNT-20% iron oxide.

The morphologies of these samples were obtained by SEM as shown in Figure 2. The diameter of the CNTs, with sponge-like structure, varied from 20 to 50 nm with an average diameter of 25 nm. The surface of CNTs after impregnation with Fe_2_O_3_ showed no surface changes and appeared to be agglomerated and untangled.

**Figure 3:**
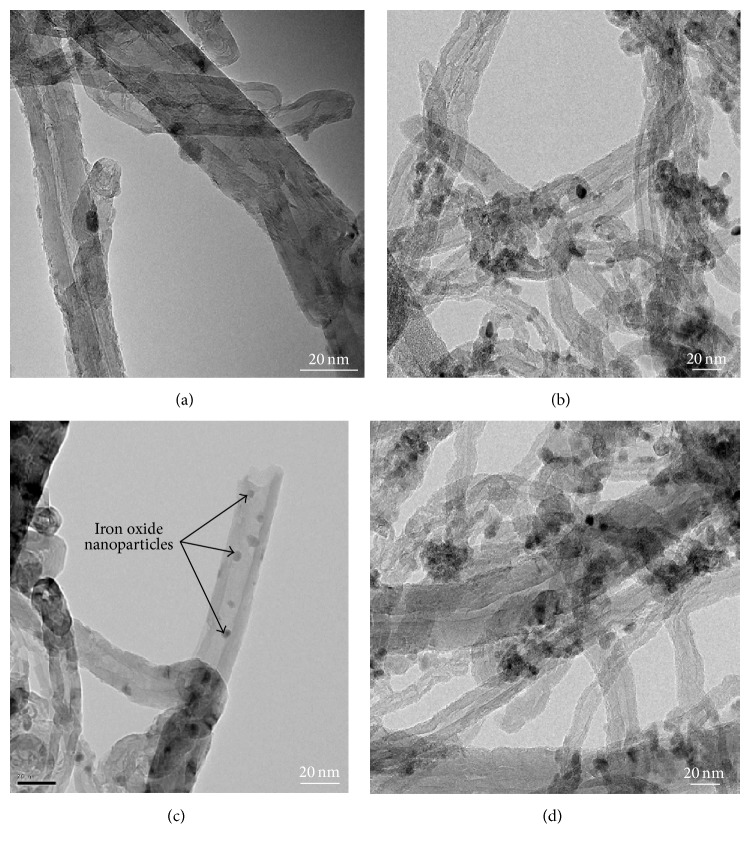
HRTEM images of (a) raw CNTs, (b) CNT-5% iron oxide, (c) CNT-10% iron oxide, and (d) CNT-20% iron oxide.

High resolution transmission electron microscopy (HR-TEM) was carried out to characterize the size, structure, and purity of the iron oxide nanoparticles doped and virgin CNTs. The raw CNTs TEM image presented in Figure 3 clearly indicates a highly ordered CNTs crystalline structure with diameter ranging from 10 to 30 nm and length from 10 to 30 *μ*m. In addition, it can be noted that the clear fringes of the graphitic sheets are well separated by 0.35 nm and are aligned with a tilted angle of about 2° toward the tube axis. The TEM images of CNTs doped with Fe_2_O_3_ nanoparticles are presented in Figures 3(b)–3(d) in order to substantiate the presence of iron oxide nanoparticle on the surfaces of CNTs. The white iron oxide nanoparticles with some spherical and irregular shapes are shown in the TEM images. The size of Fe_2_O_3_ nanoparticles is estimated to be around 1–5 nm which are somehow distributed evenly and on some locations agglomerated slightly causing increase in the nanoparticle size.

To confirm the presence of iron oxide and also to experimentally find out the percentage of iron oxide nanoparticles on surface of CNTs, EDS analysis was conducted and the results are shown in Table 1.

**Figure 4:**
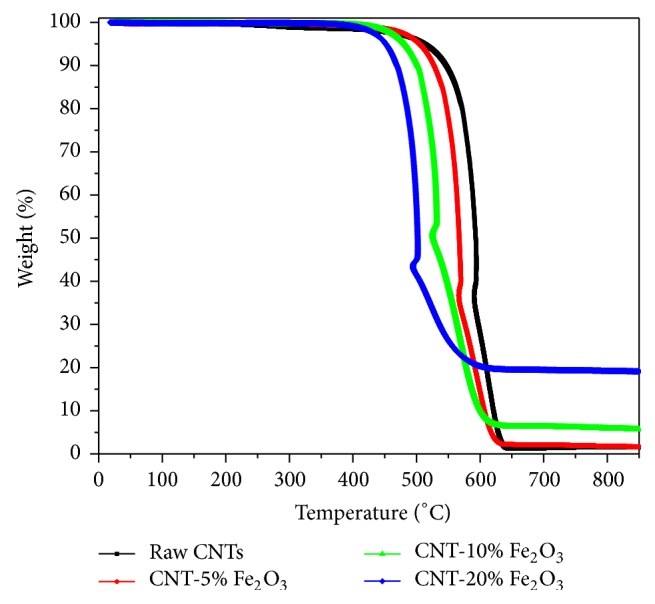
TGA analysis of raw CNTs and CNT-5% iron oxide, CNT-10% iron oxide, and CNT-20% iron oxide.

The Fe_2_O_3_ content of the impregnated CNTs was also investigated using TGA. The experiment was carried out using air at a heating rate of 10°C/min. The thermograms are shown in Figure 4. Under this operating condition, the raw CNTs decomposed and oxidized completely as verified by the TGA curve in Figure 4. As Fe_2_O_3_ content on surface of CNTs increased, higher residual yield was found which corresponded to the presence of iron oxide NPs on the surface of CNT [37]. TGA provides an accurate estimate of the loading of iron oxide NPs doped on surface of CNTs by comparing the residues for the complete oxidation of the raw and impregnated CNTs. Therefore, CNTs with 1%, 10%, and 20% Fe_2_O_3_ loading had residual yields of 6 wt.%, 8 wt.%, and 17 wt.%, respectively. Moreover it can be noted that the increase in Fe_2_O_3_ NP loading resulted in a decrease of the decomposition temperature. It can also be inferred, from the TGA analysis shown in Figure 4, that raw CNTs start to decompose at 540°C while the 5%, 10%, and 20% loaded CNTs with Fe_2_O_3_ decomposed at lower temperatures (450, 430, and 410°C, resp.). According to Chiang et al. [43] the earlier decomposition of CNTs impregnated with metallic NPs can be explained by reduction of the thermal stability of CNTs by catalyzing the low-temperature oxidation of CNTs. Therefore, it can be inferred that the presence of nanosized iron oxide particles with high surface area altered the thermal stability of the CNTs and catalyzed oxidation of impregnated CNTs under the air compared to the pristine CNTs. The nanosized metallic particles might act as a heating accelerant that progresses the heat transfer to the surface of the CNTs and enhance the oxidation process.

**Figure 5:**
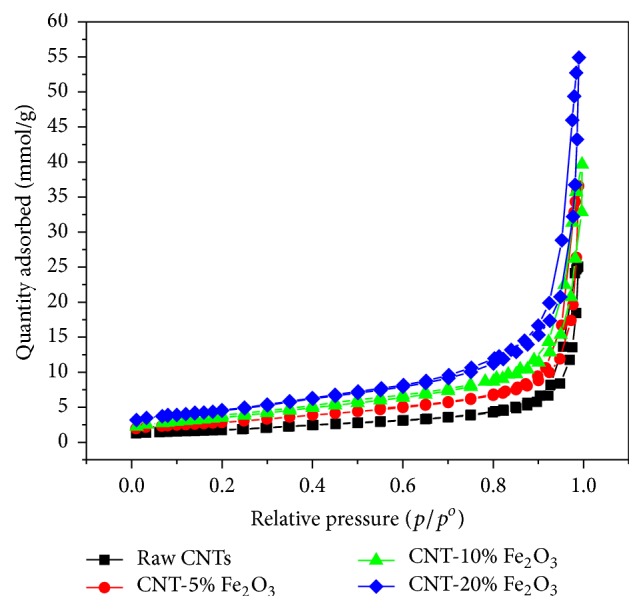
BET adsorption of N_2_ at 77 K for raw CNTs and CNT-5% iron oxide, CNT-10% iron oxide, and CNT-20% iron oxide.

The surface area of raw CNTs and impregnated CNTs was measured using BET surface area analyzer. As shown in Figure 5, the surface area of raw CNTs and CNTs impregnated with 5%, 10%, and 20% iron oxide nanoparticles was found to be 137.7, 226.6, 295.4, and 360 m^2^/g, respectively. The presence of the nanosized iron oxide particles doubled the surface area upon using 20 wt.% iron oxide nanoparticles. Increasing the surface area inherently increases the number of adsorption sites available for the removal of selenium and therefore results in higher adsorption capacity.

**Figure 6:**
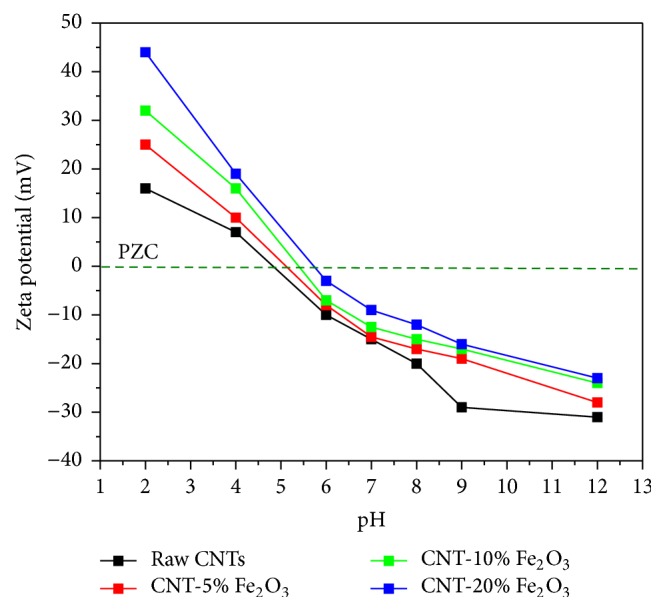
Zeta potential of raw CNTs and CNTs impregnated with 1%, 10%, and 20% Fe_2_O_3_.

Finally, the surface charge of the CNTs and impregnated CNTs with iron oxide nanoparticles was measured and the results are illustrated in Figure 6. When the Fe_2_O_3_ nanoparticles were loaded onto the surface of CNTs the negative sign of zeta potential on the surface of carbon nanotubes decreased due to neutralizing the repulsive effects of the electrical double layers. However, selenium (SeO_3_^2−^) has very large negative zeta potential (−0.37 V). As the negative sign of zeta potential decreases, the electrostatic attraction would make and attachment between CNTs and selenium ions more likely [38]. Thus, it is important to decrease the electrostatic repulsion barrier between the selenium ions and CNTs to further improve the adsorption process. It is clear from the zeta potential measurements in Figure 6 that increasing the percentage of Fe_2_O_3_ nanoparticles onto the surface of CNTs caused a reduction in the negative sign of zeta potential. The trends obtained here are in good agreement with the adsorption measurements carried out in this work, where increasing the loading of Fe_2_O_3_ nanoparticle onto the surface of CNTs enhanced the removal of the selenium ions from the water. On the other hand, loading Fe_2_O_3_ NPs onto the surface of CNTs alters the point of zero charge (PZC) of the composite. PZC is a pH value at which material has zero zeta potential. Raw CNTs have PZC at pH of 4.6 while CNTs with 5%, 10%, and 20% Fe_2_O_3_ loading have PZC at pH of 5.2, 5.6, and 5.9, respectively.

### 3.2. Effect of pH

The pH of the solution plays an important role in the adsorption of selenium ions on the adsorbent surface as it is dependent on the surface properties of the impregnated CNTs and distribution of selenium ions in water.

**Figure 7:**
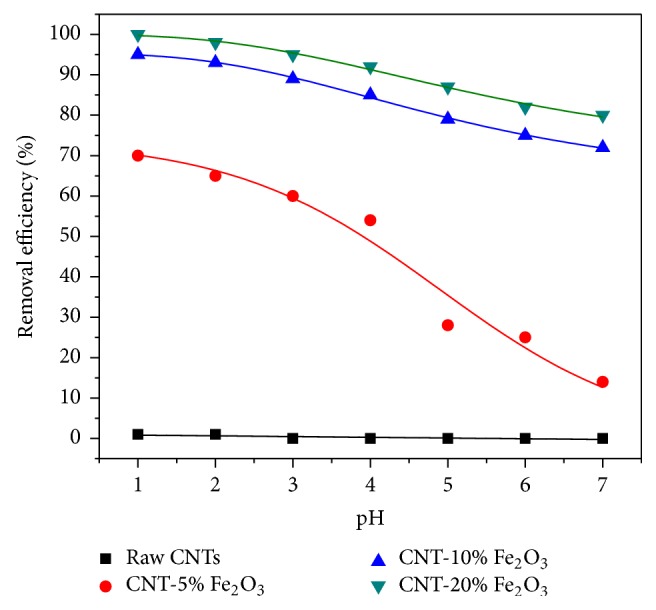
Effect of pH on the removal of selenium ions by raw and impregnated CNTs with different loading of iron oxide (agitation speed = 50 rpm, CNTs dosage = 10 mg, time = 6 hr, and initial concentration = 1 ppm).

The effect of pH on the removal of selenium ions is presented in Figure 7. The adsorption of selenium species was higher at lower pH and the removal was observed to decrease with increase in pH for the impregnated CNTs. The maximum removal was observed at pH 1. This higher removal at lower pH was due to the higher positive surface charges as indicated by zeta potential of the CNTs that favors the adsorption of anion (e.g., SeO_3_^2−^) [44]. This can be explained by release of OH ions when anion or weak acid is adsorbed onto hydroxide, which has favor toward adsorption of SeO_3_^2−^ at low pH. Zhang et al. [38] used activated carbon doped with iron oxide for the removal of selenium from water. The authors reported that selenium removal sharply dropped after pH 8 and the maximum removal occurred in acidic solutions with pH between 1 and 3. A similar trend was previously observed in case of selenium ions adsorption on iron oxide nanoparticle [8], iron-coated GAC [38], hematite [45], soil [46], hydroxyapatite [39, 47], goethite and hydrous ferric oxide [48, 49], aluminum oxide coated sand [40], metal oxide nanoparticles [50], goethite [51], nanoscale zero-valent iron [52], and cellulose microcolumn [53].

It is anticipated that at lower pH, the adsorbent surface carries a additional positive charge and the anionic species are preferably adsorbed on the surface due to electrostatic attractions [8, 46]. Monteil-Rivera et al. [47], however, reported that Se removal by hydroxyapatite increases with pH from pH 7 to 8.5, and it decreases above pH 8.5. In general, the adsorption capacity of Se was decreased slightly upon increasing the pH of solution. This might be due to the increase in the negative charge of the adsorbent surface and the consequent competition between the OH^−^ ions and selenium ions for the available adsorption sites [44, 45]. Figure 7 also reveals that raw CNTs were not efficient in removing Se ions from solution as shown in the flat trend that it depicts in Figure 6. The adsorption of selenium was less than 1% at pH between 1 and 2. Negligible (close to zero) was recorded at pH of 2. In contrast, it can be seen from the trends in Figure 7 that vast improvement on the removal of Se from solution was achieved upon impregnating the surface of raw CNTs with iron oxide.

The percentage of iron oxide loading on the CNTs surface influenced the removal rate of Se. In general, it was observed at 20% loading rate that near 100% removal of Se was achieved compared to 93% and 65% for a loading rate of 10 and 5% iron oxide, respectively, at a pH of 1 for example. A number of factors may be attributed to this marked change. For example, the attachment of iron oxide particles onto the surface of CNTs may provide ample adsorption sites for the selenium ions to interact with. Also, it is well known that surfaces of metal oxides nanoparticles in aqueous solution are covered with hydroxyl groups [18]. Therefore, anion adsorption occurs by positive adsorbent surface charge (less negative sign compared to anion). Generally, increasing the pH causes decrease in the adsorbent surface charge and, accordingly, decreases in the adsorbent capacity [18, 39, 54]. As a result, when pH is increased the adsorbent surface is negatively charged (more negative sign) and leads to repulsion between negatively charged adsorbent particles and selenium anions. This repulsion causes termination of the adsorption process and also leads to the release of adsorbed selenium anions on surface of CNTs at higher pH to the water (desorption process). Based on the results reported in Figure 7, CNTs impregnated with 20 wt.% of iron oxide were selected in the later experiments to study the effects of other variables such as the initial Se concentration, CNTs dosage, contact time, and kinetics and isotherms models.

### 3.3. Effect of Initial Concentration

**Figure 8:**
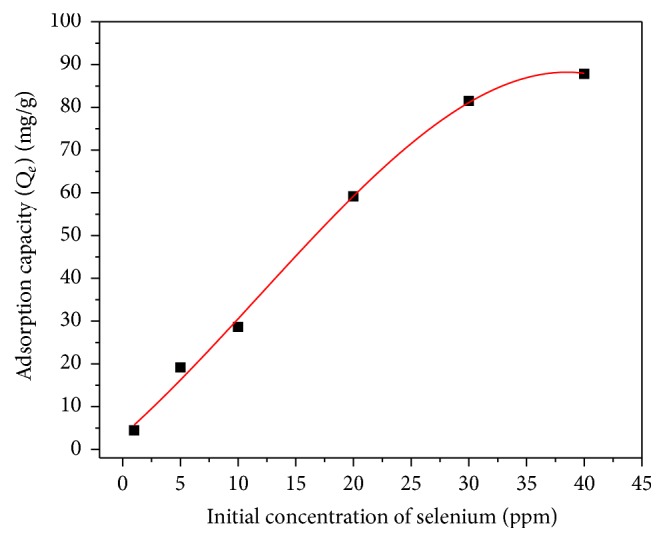
Effect of initial concentration of selenium on adsorption capacity of CNTs-20% iron oxide (agitation speed = 150 rpm, pH = 6, CNTs dosage = 10 mg, and time = 6 hr).

Figure 8 depicts the impact of initial Se concentration on the adsorption capacity of iron oxide impregnated CNTs. In general, the trend showed a marked increase of adsorption capacity with the increase in Se initial concentration. Figure 8 shows that at Se initial concentration between 5 and 20 ppm, the adsorption rate was steep and the adsorption was fast as can be seen from the slope of the first trend line. Above 20 pmm the adsorption slowed down and a plateau (second trend line) can be observed. The decline in the rate of adsorption at initial concentrations higher (as can be seen from the slope of the trend line) than 30 ppm (Figure 8) may be attributed to the saturation of all adsorption sites on the surface of CNTs. The higher adsorption capacity at higher Se concentration may be due to increase in the mass transfer (driving force) of selenium ions towards the iron oxide impregnated CNTs surfaces [18]. The highest adsorption capacity was about 88 mg/g at an initial Se concentration of 40 ppm as shown in Figure 8.

### 3.4. Effect of CNTs Dosage

**Figure 9:**
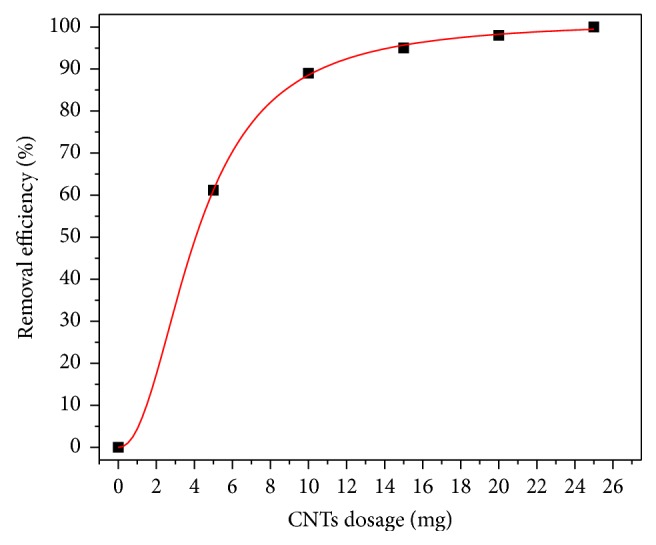
Effect of CNTs-20% iron oxide dosage on selenium removal (agitation speed = 150 rpm, pH = 6, time = 6 hr, and initial concentration = 1 ppm).

The amount of CNTs added to the solution was varied between 5 and 25 mg in order to study the required optimum amount of adsorbent required to carry out the adsorption duty. In the experiments, the contact time, agitation speed, and pH were kept constant at 6 hr, 150 rpm, and 6, respectively. The experimental results are shown in Figure 9. Adsorption percentage (%) of selenium was plotted as a function of adsorbent dosage. Selenium ions adsorption was increased with increasing CNTs dosage due to the increase in the adsorption sites on CNTs surfaces resulting in the increase amount of adsorbed selenite ions. Selenium ions were completely removed from the solution using only 25 mg of CNTs. Therefore, the results show that the impregnated CNTs were suitable to adsorb selenium ions completely when there was sufficient CNTs surface area in the solution. Although the data was not shown here, the adsorption capacity was high at low dosages and keeps reducing at higher dosages. It is clearly agreed that as adsorbent dosage increases, the number of available adsorption sites increases, too. Alternatively, the decrease in adsorption capacity with increase in the adsorbent dosage is mostly related to the unsaturated nature of the adsorption sites through the adsorption process.

### 3.5. Effect of Contact Time

**Figure 10:**
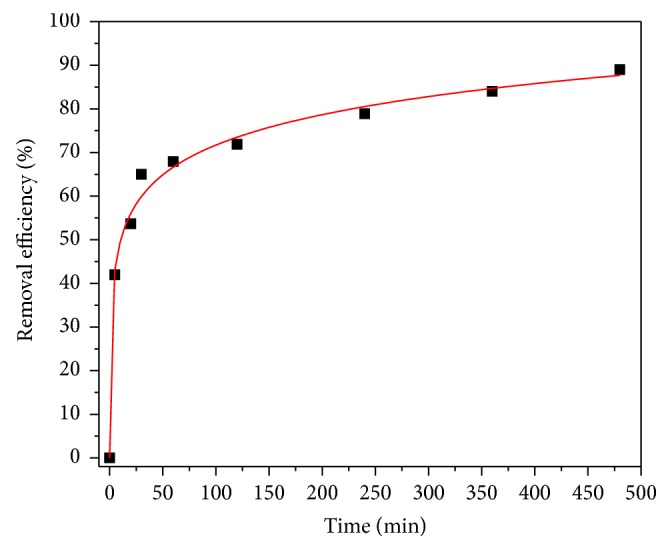
Effect of contact time on selenium removal (agitation speed = 150 rpm, pH = 6, CNTs dosage = 10 mg, and initial concentration = 1 ppm).

The effect of contact time on the adsorption of selenium ions is presented in Figure 10. It can be clearly seen that the rate of adsorption of selenium increased at initial period of contact time and then it decreased gradually with time until the adsorption reached an equilibrium point. The adsorption of selenium has increased rapidly during the first 30 minutes to reach about 65% removal. After that a slight increase was observed in the adsorption to reach the maximum removal of selenium within four hours. The fast rate and then slow rate of adsorption suggest that selenium ions were first adsorbed on exterior surfaces of the CNTs during the initial time of contact. When the exterior surface gets saturated, the selenium ions diffused into the pores of the CNTs and were adsorbed at the interior surface of the CNTs.

### 3.6. Adsorption Kinetic Models

**Figure 11:**
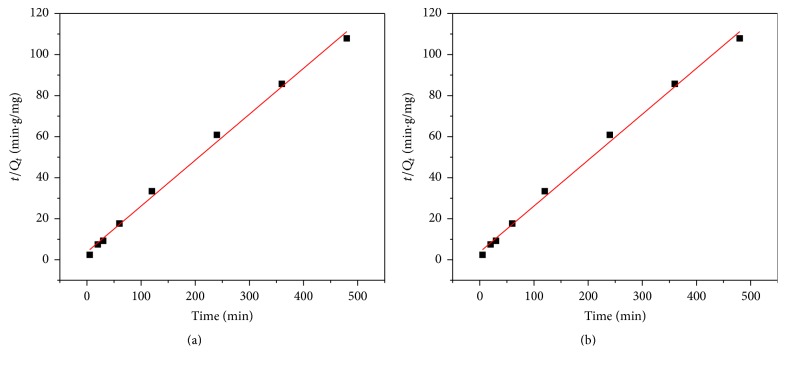
Adsorption kinetic for selenium removal: (a) pseudo-first-order and (b) pseudo-second-order model (agitation speed = 150 rpm, pH = 6, CNTs dosage = 10 mg, and initial concentration = 1 ppm).

The modeling of the kinetics for selenium adsorption on CNTs was investigated using pseudo-first-order and pseudo-second-order kinetic models. Linearized plots of the two models are shown in Figure 11.

From Figure 11, the selenium adsorption on Fe_2_O_3_ impregnated CNTs does not fit very well to the pseudo-first-order model, as the *R*^2^ values are less than 0.5. On the other hand, it does fit perfectly the pseudo-second-order model with *R*^2^ value being almost 1. The rate constant for the 20% Fe_2_O_3_ loaded CNTs is found to be 0.016 g/mg·h.

The result obtained in this study is in good agreement with those reported in literature as most of the solid-liquid adsorption processes tend to conform to the pseudo-second-order model. Moreover, the pseudo-second-order model in this study can be defended by two-step linear relationship supporting the chemisorption nature of the process which is considered as rate-controlling mechanism as well [55]. The two-step linear mechanism between the composite CNTs (indicated as C and XC as activate adsorption site on CNTs) and Se ions is as follows:

(8) 2C+Se2+⟶Se2XC+Se2+⟶SeC2+2X+.

As it was found in Figure 11, the adsorption is pseudo-second-order and the rate-limiting step is the chemical adsorption between Se ions and surface of CNTs through sharing or the exchange of electrons. Similar trends are reported for selenium ion adsorption in literature where the adsorption mechanism fit to pseudo-second-order model [38, 39].

### 3.7. Adsorption Isotherms Models

**Figure 12:**
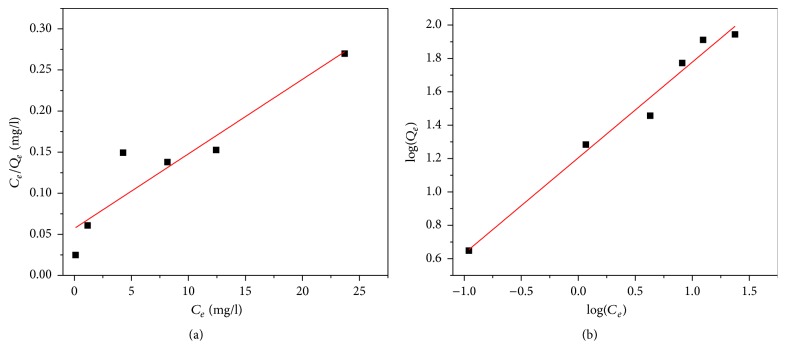
Adsorption isotherm models of selenium: (a) Langmuir and (b) Freundlich.

The adsorption data is modeled using the Freundlich and Langmuir isotherm models. As shown in Figure 12, the ability of the Freundlich model to fit the experimental data was studied by generating a plot of ln⁡*Q*_*e*_ versus ln⁡*C*_*e*_ with the intercept value of *K*_*f*_ and the slope of *n*. It can be observed from Table 3 and Figure 12 that Freundlich isotherm model best fit the data (*R*^2^ = 0.98). From Figure 12(a) the Freundlich constants *K*_*f*_ and *n* for the CNTs were found to be 16 and 1.74, respectively. The value of *n* or slope of the fit is an indication of sorption intensity or surface heterogeneity. When the slope gets closer to zero, the system tends to become more heterogeneous and a value of the slope greater than unity implies a favorable process and indicative of cooperative sorption. Therefore, as value of n increases, the sorption process between selenium ion and CNTs is more favorable and this means there is better bonding between sorbent and selenium. However, the Langmuir isotherm model (Figure 12(a)) fit reasonably *R*^2^ value of 0.879. The maximum adsorption capacity of the iron oxide impregnated CNTs, as predicted by isotherm model, was found to be 111 mg/g.

Interestingly, composite made of CNTs and iron oxide nanoparticle showed very superior adsorption capacity compared to each one of the mentioned adsorbent in Table 2, where the capacity could reach maximum of 111 mg/g for selenium removal. There are few factors which contributed to the very high adsorption capacity compared to other materials in Table 3. Superior surface area, high zeta potential, and special surface structure of CNTs suggest that CNTs have great potential for use as contaminant adsorbents in wastewater treatment. The only limiting factor which might affect usage of CNTs in the application such as environmental protection or water treatment is their production cost. It is not very far if we predict that the cost will further reduce by advancement in technology and CNTs then could be one of the potential for application such as water treatment.

## 4. Conclusions

The study revealed that CNTs impregnated with 20 wt.% of iron oxide showed 100% removal of selenium ions in 6 hours with an initial concentration of 1 ppm at pH 6, adsorbent dosage of 25 mg, and agitation speed of 150 rpm. The adsorption data very well fitted to the Freundlich model with maximum adsorption capacity of the iron oxide impregnated CNTs predicted by Langmuir isotherm model to be 111 mg/g. The date was correlated very well to pseudo-second-order kinetic model and rate of constant was found to be 0.016 g/mg·h. The highest adsorption capacity of iron oxide impregnated CNTs suggested that it can be employed effectively for the removal of selenium ions from water.

## Figures and Tables

**Table 1:** EDS analysis of raw CNTs and CNTs with different percentage of Fe_2_O_3_.

CNTs sample	Raw CNTs	CNT-Fe_2_O_3_ (5%)	CNT-Fe_2_O_3_ (10%)	CNT-Fe_2_O_3_ (20%)
Element	Weight %	Weight %	Weight %	Weight %
C	98.50	91.80	84.53	56.4
O	1.50	3.65	4.44	25.52
Fe	0	4.55	11.03	18.08
Total %	*100*	*100*	*100*	*100*

**Table 2:** Parameters of Langmuir and Freundlich adsorption isotherm models of selenium.

Langmuir			Freundlich		
*Q*_*m*_ (mg/g)	*K*_*L*_ (Lmg^−1^)	*R* ^2^	*n*	*K*_*f*_ (mg^(1−1/*n*)^L^1/*n*^g^−1^)	*R* ^2^
111	0.158	0.879	1.74	16	0.98

**Table 3:** Adsorption capacity of different materials for adsorption of selenium from water.

Type of adsorbent	Experimental conditions	Max. adsorption capacity (mg/g)	Reference
Iron oxide nanoparticle	pH = 4, initial concentration = 0.01 mg/L	15.1	[8]
Chitosan–clay composite	pH = 4, initial concentration = 0.1 mg/L, *T* = 30°C	18.4	[15]
Iron-coated GAC	pH = 2–8, initial concentration = 2 mg/L, *T* = 45°C	2.89	[38]
Nanocrystalline hydroxyapatite	pH = 5, initial concentration = 0.01 mg/L, *T* = 30°C	1.94	[39]
Aluminum oxide coated sand	pH = 4.80, initial concentration = 1.2 mM	1.08	[40]
Sulfuric acid treated rice husk	pH = 1.5, initial concentration = 100 mg/L *T* = 45°C	40.92	[41]
Iron oxide impregnated CNTs	pH = 6, CNTs dosage = 10 mg and initial concentration = 1 ppm	111	This study
